# Technical Note: PYRO‐NN: Python reconstruction operators in neural networks

**DOI:** 10.1002/mp.13753

**Published:** 2019-08-27

**Authors:** Christopher Syben, Markus Michen, Bernhard Stimpel, Stephan Seitz, Stefan Ploner, Andreas K. Maier

**Affiliations:** ^1^ Pattern Recognition Lab Friedich‐Alexander Universität Erlangen‐Nürnberg 91058 Erlangen Germany

**Keywords:** inverse problems, known operator learning, machine learning, open source, reconstruction

## Abstract

**Purpose:**

Recently, several attempts were conducted to transfer deep learning to medical image reconstruction. An increasingly number of publications follow the concept of embedding the computed tomography (CT) reconstruction as a known operator into a neural network. However, most of the approaches presented lack an efficient CT reconstruction framework fully integrated into deep learning environments. As a result, many approaches use workarounds for mathematically unambiguously solvable problems.

**Methods:**

PYRO‐NN is a generalized framework to embed known operators into the prevalent deep learning framework Tensorflow. The current status includes state‐of‐the‐art parallel‐, fan‐, and cone‐beam projectors, and back‐projectors accelerated with CUDA provided as Tensorflow layers. On top, the framework provides a high‐level Python API to conduct FBP and iterative reconstruction experiments with data from real CT systems.

**Results:**

The framework provides all necessary algorithms and tools to design end‐to‐end neural network pipelines with integrated CT reconstruction algorithms. The high‐level Python API allows a simple use of the layers as known from Tensorflow. All algorithms and tools are referenced to a scientific publication and are compared to existing non‐deep learning reconstruction frameworks. To demonstrate the capabilities of the layers, the framework comes with baseline experiments, which are described in the supplementary material. The framework is available as open‐source software under the Apache 2.0 licence at https://github.com/csyben/PYRO-NN.

**Conclusions:**

PYRO‐NN comes with the prevalent deep learning framework Tensorflow and allows to setup end‐to‐end trainable neural networks in the medical image reconstruction context. We believe that the framework will be a step toward reproducible research and give the medical physics community a toolkit to elevate medical image reconstruction with new deep learning techniques.

## 1 Introduction

In recent years, major breakthroughs made deep learning increasingly prevalent in more and more fields. It revolutionizes the way of classification and regression tasks in speech and image recognition^1^, ^2^, ^3^ and many other areas. Even in the medical domain, where interpretability and reliability are one of the most important driving forces, deep learning has led to astonishing results.^4^ One of the most cited papers of recent years is the U‐net^5^ which outperforms classical machine learning algorithms in segmentation tasks. In the subsequent time, the U‐net architecture emerged to many more tasks, for example, artifact correction, image fusion, image‐to‐image translation, and even into the context of medical image reconstruction.^6^, ^7^, ^8^ However, this domain is fundamentally different from those in which the advent of deep learning began, and the question arises as to whether these learned signal reconstruction pipelines are reliable and stable enough for a critical area such as medical imaging.^9^ Two special issues: “*Deep learning in medical imaging*”^10^ and “*Machine Learning for Image Reconstruction*”^11^ in transactions on medical imaging (TMI) in 2016 and 2018 discuss the increasing relevance of deep learning methods in medical image reconstruction.

The presented approaches can be divided into either pre‐ or post‐processing approaches or fully end‐to‐end trained methods. For the first type, the actual reconstruction pipeline is based on well known signal reconstruction algorithms omitting the end‐to‐end capability due to its complexity. For the second type of approaches, the modeling of the end‐to‐end pipeline can be realized under two different paradigms. One way is to learn the whole signal processing pipeline, an exceptionally clear representative of this paradigm is *AUTOMAP*.^12^ Directly in contrast to this is the emerging paradigm of embedding known operators.^13^ This preserves the end‐to‐end learning capability but includes the known operations of the reconstruction chain to preserve the credibility of the signals, reduce the error bound of the learning process and decrease the number of parameters and thus the amount of necessary training data. This paradigm gets increasingly popular, with multiple publications following the way of embedding known operators in the computed tomography (CT) context and successfully including the CT reconstruction as known operators into the network architecture to be able to benefit from the end‐to‐end training capability of deep learning.^14^, ^15^, ^16^, ^17^, ^18^, ^19^, ^20^, ^21^ However, the publications that follow this path are still less represented than those that use deep learning only as pre‐ or post‐processing. We believe that a major reason for this is the non‐trivial implementation of known operators in existing deep learning frameworks. Even publications that successfully take on this challenge often refer to their own implementations as prototypical^15^ or provide frameworks on abstract wrapped levels.^16^, ^22^ An efficient and publicly usable solution integrated into one of the popular deep learning frameworks, however, remains pending.

To strengthen the paradigm of known operators, elaborate the research in the medical image reconstruction, and to avoid reimplementations and incompatibilities, we started to work on an open source software framework PYRO‐NN, which allow an easy way to integrate known algorithms into the deep learning framework Tensorflow.^23^ We provide multiple forward and backward projectors for CT implemented in CUDA based on scientific publications supported with a high‐level Python API for simple use of state‐of‐the‐art CT reconstruction, even from different setups of real CT scanners. The profound integration into Tensorflow on C++/Cuda level allows to handle occurring performance and memory issues and, additionally, allows an easy customization of the algorithms compared to a wrapper alternative like.^22^, ^24^ Furthermore, the high‐level Python API offers an easy link between deep learning and community driven frameworks. For the CT domain this allows to use a wide range of tools (e.g., filter, redundancy weights, etc.).^24^, ^25^, ^26^, ^27^

We believe that this framework will help the community leverage the power of end‐to‐end training of machine learning algorithms directly from the data, while continuing to apply mathematically sound solutions to uniquely solvable problems.

## 2 Materials and Methods

The framework concept is designed to include native C++ and CUDA based algorithms into the deep learning framework Tensorflow. In detail, PYRO‐NN provides network layers as CUDA implementations to generate parallel‐, fan‐, and cone‐beam x‐ray projections and to reconstruct them within any neural network constructed with Tensorflow. Due to the nature of the projection and reconstruction operation, we intrinsically provide the analytical gradients for all of these layers with respect to their inputs, which allows fully end‐to‐end trainable networks. Furthermore, with PYRO‐NN we provide filters and weights based on scientific publications to allow proper filtered‐backprojection (FBP) reconstructions. The PYRO‐NN API is inspired by the CONRAD^26^ framework to adapt the ability to reconstruct data from real clinical scanners and by using PyConrad^27^ many more tools and phantoms can easily be used in the deep learning context. The current state of the framework features a CT reconstruction pipeline, while the basic design allows to transfer the whole concept to other signal reconstruction domains within one framework and, therefore, points out a direction to future development and community contribution.

### 2.A. Software design/rationale

The development speed in the deep learning community is tremendous. Like in the research itself, the toolkits and frameworks are developing in the same speed, which often causes conflicts in interoperability of self‐developed solutions and version mismatches between different frameworks and toolkits. To ensure a robust version control, the framework is directly included into the building process of the Tensorflow sources.

#### 2.A.1. PYRO‐NN‐layers

The known operators can be implemented as CUDA kernels with an additional C++ class following the design of the Tensorflow API for the embedding as a Tensorflow layer. Unlike other frameworks that simply wrap the implementation at the Python level, this provides the advantage of full control over device resources such as memory utilization and implementation efficiency. The separation of the operator implementation as a native CUDA kernel and the information control allows an easy extension towards other deep learning frameworks. The integration of PyTorch is planned for the future. The integration of known operators can be found under: https://github.com/csyben/PYRO-NN-Layers.

#### 2.A.2. PYRO‐NN

We provide a high‐level Python API to allow a convenient use of the known operators as normal Tensorflow layers and offers additional helper functions. The provided Python package automatically invokes the relevant algorithms to compute the gradient with respect to the input of the layer in an efficient way. The provision of the gradient is a necessity to enable a gradient flow through the entire network and, thus, allow fully end‐to‐end trainable networks with known operators. The package can be installed via *pip* or from: https://github.com/csyben/PYRO-NN.

All together, these rationales offer the community with a generic, version stable, framework to easily include known operators into neural networks. The source code is publicly available under the Apache 2.0 licence to be directly compatible with Tensorflow and to allow uncomplicated community contributions to existing projects. A detailed description of the software architecture and the build process can be found in the supplementary material Section 1.

### 2.B. CT reconstruction in neural networks

Based on the generic design of the framework, the current state provides all necessary algorithms and tools for analytical parallel‐, fan‐, and cone‐beam reconstruction. The necessary algorithms are implemented within Tensorflow as an own layer, while the respective tools, for example, filter, weights, etc., are provided on the Python level to supply a high‐level API for CT reconstruction. In the following, we introduce the mapping of the known operator to a layer for our case study of CT reconstruction, followed by a description of the provided algorithms and tools.

#### 2.B.1. The known operator

For the task of reconstructing object information from acquired x‐ray projections, an efficient analytical method is well known and is called FBP. To embed these methods into a neural network, the whole acquisition and reconstruction procedure of a CT system needs to be described with discrete linear algebra to embed them into a neural network. The acquisition of projection data of the object can be described with

(1) Ax=p,

where **A** is the matrix describing the geometry, the so called system matrix which can be algorithmically implemented as the forward‐projection operator. The object is denoted by **x** and **p** are the acquired projections of object **x** under the system described by **A**. The reconstruction according to the FBP algorithm can be conducted using the Moore‐Penrose pseudoinverse for the system matrix $A^{⊤}{\left({\mathrm{AA}}^{⊤}\right)}^{-1}$ which gives:

(2) $$x=A^{⊤}F^{H}KFp,$$

where $A^{⊤}$ is the adjoint system matrix which can algorithmically implemented as the back‐projection operator. According to the FBP, the inverse bracket describes a filter operation, which is conducted by a multiplication with the diagonal filter matrix **K** in the Fourier domain. Consequently **F**, $F^{H}$ is the Fourier transform and the respective adjoint, that is, inverse operation. Hence, the forward and backward model can be expressed completely as discrete linear algebra, allowing fully end‐to‐end trainable networks. As the publications from Würfl and Syben et al. show that **A** and $A^{⊤}$ are their respective operators to calculate the gradient, therefore the gradient flow through these layers can be ensured.^17^, ^19^

#### 2.B.2. The operator as a layer

From iterative reconstruction, it is known that the system matrix is usually too large to store in memory; therefore, we compute the operator on the fly using ray‐based algorithms. There are several ways for the computation. We introduce the *ray‐driven forward‐projection* and the *voxel‐driven back‐projection* algorithmically as native CUDA kernels for the integration into Tensorflow. Note that when using a ray‐driven forward‐projection algorithm to compute the result of the multiplication with **A**, then the voxel‐driven back‐projection algorithm is not the respective adjoint operation $A^{⊤}$. They are a so called an unmatched projector‐/back‐projector pair. The implications of matched projectors and shear‐warp projectors on the convergence and runtime are subject to future work and are briefly discussed in Section 3.

The *forward‐projection* to generate projections from the input volume are implemented as CUDA kernels in a ray driven manner. For each detector pixel, a ray $\vec{r}$ is cast through the scene, accumulating the absorption values along the line. We provide forward projectors for two‐dimensional (2D) parallel‐ and fan‐beam geometry based on ray vectors and respective geometry parameters. Furthermore, a three‐dimensional (3D) cone‐beam forward projector based on projection matrices is implemented according to Galigekere et al.^28^ The CUDA kernels are parallelized over the detector pixels computing the line integral along the ray.

The *back‐projection* operators to reconstruct simulated or real projection data are implemented as CUDA kernels in a voxel‐driven manner. For each pixel/voxel to be reconstructed, the projection of the point on all projection images is accumulated. The framework provides the respective 2D parallel‐ and fan‐beam back‐projection algorithms based on geometry parameters and ray vectors. Following the forward projection, the 3D cone‐beam back‐projection is based on projection matrices according to Scherl et al.^29^ Note that for runtime efficiency, the distance weighting for the cone‐beam circular trajectory geometry is included within the kernel. Currently, only circular trajectories are supported by default. Thus, we recommend adapting the projectors for special reconstruction accordingly. The back‐projection kernel is parallelized over the voxels projecting the respective position on the different detector coordinates interpolating the measured line integral.

For the 3D cone‐beam case, the framework offers the possibility to choose between a texture and a kernel interpolation mode. While texture interpolation is associated with very short computing times, Tensorflow’s memory management in combination with CUDA implies that the data must be kept twice in memory. For kernel interpolation, the situation is exactly the other way around, the computations are slower but no additional memory is needed. As both options are provided the user can decide on a per application bases. Furthermore, as the 3D cone‐beam operators are based on projection matrices, calibrated matrices from real systems can be used as shown in the CONRAD framework.^26^

### 2.C. High‐level python API

To supply the community with an easy‐to‐use version of the described layers, we provide the necessary structure and additional tools like filters, weights, phantoms, etc., within the Python framework. In the following, the outline of the necessary structure to utilize the layers is shown, followed by a short introduction of the provided tools.

#### 2.C.1. Reconstruction and geometry

The high‐level Python API wraps the provided reconstruction layers in Tensorflow. Thus, the framework registers the respective adjoint operation for the gradient computation automatically. All attributes necessary for the provided forward‐projection and backward‐projection layers are covered with a base geometry class and corresponding specialized derived geometry classes, for example, cone‐beam geometry class dependent on projection matrices.

#### 2.C.2. Phantoms

PYRO‐NN contains a set of simple geometric objects, for example, circle, ellipsoid, sphere, and rectangles to easily create a more complex numerical phantom. Furthermore, the framework provides an analytical description of the 2D Shepp–Logan phantom^30^ as well as a 3D extension of it based on the CONRAD implementation.^26^

#### 2.C.3. Trajectories

The trajectory describes the geometric scanner setup over the whole scan. For the 2D parallel‐ and fan‐beam cases, the trajectory is described by the central ray vector for each projection. For the 3D cone‐beam case, the trajectory is described by a set of projection matrices, which allows to use calibrated projection matrices from real scanner systems. Within the high‐level Python API, we provide basic methods to compute the respective rays or projection matrices based on a given geometry. The open‐source concept of the whole framework allows to contribute to the diversity of provided trajectories.

#### 2.C.4. Filters

To allow a basic reconstruction in the context of neural networks, PYRO‐NN provides the Ramp and Ram‐Lak filter implemented according to Kak and Slaney.^31^ The filters can be directly assigned as weights to a multiplication layer and are a multiplication with a diagonal matrix in the Fourier domain as shown in Eq. (2).

#### 2.C.5. Correction weights

In order to support fan‐ and cone‐beam reconstructions for the short‐scan case, the framework contains geometric and redundancy correction weights implemented according to Kak and Slaney.^31^

#### 2.C.6. Network architectures

**Figure 1:**
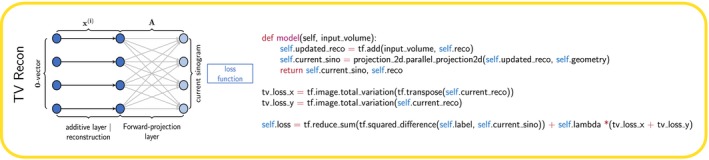
PYRO‐NN iterative reconstruction network. The training procedure solves: $\text{min}\;||Ax-{p||}_{2}^{2}+\lambda \mathrm{TV}\left(x\right)$. The seeked reconstruction is achieved when the optimal training state is reached. [Color figure can be viewed at http://wileyonlinelibrary.com]

Following the paradigm of precision learning,^13^ different network architectures can be setup or even derived as shown by Syben et al.^21^ We provide various examples within the framework to assist users in using the framework. The experiments in the supplementary material cover a baseline network able to reconstruct a short‐scan cone‐beam CT according to the Feldkamp–Davis–Kress (FDK) algorithm^32^ (Section 2.A), a reconstruction with raw‐data and projection matrices from a real system (Section 2.B), an example of learning the correct reconstruction filter discretization (Section 2.C) and a novel baseline network to perform iterative reconstruction within few lines of code (Fig. 1; Section 2.D). A detailed description of the experiments can be found in the supplementary material Section 2.A–2.D. In addition, executable experiments are made available online as a Code Ocean Capsule.^33^

## 3 Discussion

Recently, there have been several different attempts to transfer the astonishing capability of deep learning into the field of medical image reconstruction. In order to transfer deep learning toward medical image reconstruction and at the same time address these problems, the idea of embedding known operators into the neural networks is increasingly pursued as the growing number of publications shows.

In this paper, we present a framework providing the known operators for CT reconstruction and all necessary tools to conduct experiments on real scenarios. We believe that such an open‐source framework will reduce the barriers of such approaches and will elevate the research in the medical image reconstruction domain. To encourage the research, we provide baseline experiments in the supplementary material and example code within the framework, allow an starting point for own research ideas.

To the best of our knowledge, PYRO‐NN is the first framework that provides CT reconstruction algorithms as native CUDA kernels within neural networks. This allows full control over the device resources in contrast to CT algorithms wrapped on Python level. We choose to implement the projector and back‐projector as a unmatched projector pair. The implications of unmatched pairs are already analyzed in the context of iterative reconstruction by Zeng et al.^34^ Zeng et al. concluded that unmatched‐pairs can be beneficial due to the algorithmic speedup, while the convergence of the algorithm has to be kept in mind. While we have not noticed negative effects on the training process in our experiments,^19^, ^21^ we want to investigate the implications of unmatched‐projector pairs to the training procedure in future work.

As the combination of deep neural networks and CT reconstruction can, especially in the 3D case, easily exceeds the GPU’s memory, the provided algorithms allow the user a trade‐off choice between computational‐ and memory efficient implementations. Furthermore, the concept of the framework enables a problem‐specific solution, since the algorithms and the gradients can be changed by the user at any time. Additionally, as the core of PYRO‐NN is an extension of the existing Tensorflow build process, every known operator which allows the calculation of sub‐gradients can easily be modeled as a Tensorflow layer. Besides the actual CUDA implementation, there is only the need of an information control class following the Tensorflow API guidelines. Therefore, the setup allows an easy extension toward other frameworks like PyTorch as the CUDA kernel implementation of the known operator stays untouched.

We provide the known operators for CT reconstruction on CUDA level with the respective necessary tools like filters and weights on Python level. Nevertheless, the framework design allows an easy extension to other fields, for example, magnetic resonance imaging (MRI) and many more. With the increasing amount of publications being supplemented by open‐source reference implementations, we believe that with help of the community PYRO‐NN can grow beyond the application on CT reconstruction.

## 4 Conclusion and Outlook

PYRO‐NN is an open‐source software framework developed to elevate the use of known operators within neural networks. This allows to transfer the power of deep learning to medical image reconstruction while making use of existing knowledge about the physical principles. Currently, the framework provides state‐of‐the‐art CT reconstruction algorithms within the Tensorflow deep learning environment, supported by the necessary tools for the reconstruction pipeline. This allows to use existing CT reconstruction algorithms in combination with neural networks in an end‐to‐end trainable fashion. The generic design of the framework makes it easy to extend it to other modalities. We hope that our open‐source framework will encourage other groups to join these efforts making the framework a valuable element in the deep learning medical image reconstruction field. The main objective of the framework is to enable the community to use CT reconstruction algorithms in end‐to‐end neural networks and to elevate the research in medical image reconstruction. The software package is available under https://github.com/csyben/PYRO-NN and https://github.com/csyben/PYRO-NN-Layers.

## Supporting information


**Appendix S1:** Supplementary Material.

## References

[1] LeCun Y , Bengio Y , Hinton G. Deep learning. Nature. 2015;521:436. 26017442 10.1038/nature14539

[2] Krizhevsky A , Sutskever I , Hinton GE . Imagenet classification with deep convolutional neural networks In: Pereira F, Burges CJC, Bottou L, Weinberger KQ, eds. Advances in Neural Information Processing Systems 25, Red Hook, NY: Curran Associates, Inc.; 2012:1097–1105.

[3] Van Den Oord A , Dieleman S , Zen H , et al. Wavenet: A generative model for raw audio. CoRR abs/1609.03499; 2016.

[4] Maier A , Syben C , Lasser T , Riess C . A gentle introduction to deep learning in medical image processing. arXiv preprint arXiv:1810.05401; 2018. 10.1016/j.zemedi.2018.12.003 30686613

[5] Ronneberger O , Fischer P , Brox T . U‐net: Convolutional networks for biomedical image segmentation. In: *International Conference on Medical image computing and computer‐assisted intervention* Springer; 2015:234–241.

[6] Stimpel B , Syben C , Würfl T , Mentl K , Dörfler A , Maier A . MR to x‐ray projection image synthesis. arXiv preprint arXiv:1710.07498; 2017.

[7] Jin KH , McCann MT , Froustey E , Unser M. Deep convolutional neural network for inverse problems in imaging. IEEE Trans Image Process. 2017;26:4509–4522. 28641250 10.1109/TIP.2017.2713099

[8] Kofler A , Haltmeier M , Kolbitsch C , Kachelrieß M , Dewey M. A u‐nets cascade for sparse view computed tomography. In: *International Workshop on Machine Learning for Medical Image Reconstruction* Springer; 2018:91–99.

[9] Antun V , Renna F , Poon C , Adcock B , Hansen AC. On instabilities of deep learning in image reconstruction‐does ai come at a cost?. arXiv preprint arXiv:1902.05300; 2019.

[10] Greenspan H , Van Ginneken B , Summers RM. Guest editorial deep learning in medical imaging: overview and future promise of an exciting new technique. IEEE Trans Med Imaging. 2016;35:1153–1159.

[11] Wang G , Ye JC , Mueller K , Fessler JA. Image reconstruction is a new frontier of machine learning. IEEE Trans Med Imaging. 2018;37:1289–1296. 29870359 10.1109/TMI.2018.2833635

[12] Zhu B , Liu JZ , Cauley SF , Rosen BR , Rosen MS. Image reconstruction by domain‐transform manifold learning. Nature. 2018;555:487–492. 29565357 10.1038/nature25988

[13] Maier A , Schebesch F , Syben C. Precision learning: Towards use of known operators in neural networks. in 2018 24th ICPR, IEEE; 2018:183–188.

[14] Ye JC , Han Y , Cha E. Deep convolutional framelets: a general deep learning framework for inverse problems. SIAM J Imaging Sci. 2018;11:991–1048.

[15] Chen H , Zhang Y , Chen Y , et al. LEARN: learned experts assessment‐based reconstruction network for sparse‐data CT. IEEE Trans Med Imaging. 2018;37:1333–1347. 29870363 10.1109/TMI.2018.2805692 PMC6019143

[16] Adler J , Öktem O. Learned primal‐dual reconstruction. IEEE Trans Med Imaging. 2018;37:1322–1332. 29870362 10.1109/TMI.2018.2799231

[17] Würfl T , Ghesu FC , Christlein V , Maier A. Deep learning computed tomography. In: *International Conference on Medical Image Computing and Computer‐Assisted Intervention* Springer; 2016:432–440.

[18] Hammernik K , Würfl T , Pock T , Maier A. A deep learning architecture for limited‐angle computed tomography reconstruction In: Bildverarbeitung für die Medizin 2017, Berlin: Springer; 2017:92–97.

[19] Syben C , Stimpel B , Breininger K , et al. Precision learning: reconstruction filter kernel discretization. In Proceedings of the Fifth International Conference on Image Formation in x‐Ray Computed Tomography; 2018:386–390.

[20] Würfl T , Hoffmann M , Christlein V , et al. Deep learning computed tomography: learning projection‐domain weights from image domain in limited angle problems. IEEE Trans Med Imaging. 2018;37:1454–1463. 29870373 10.1109/TMI.2018.2833499

[21] Syben C , Stimpel B , Lommen J , Würfl T , Dörfler A , Maier A. Deriving neural network architectures using precision learning: Parallel‐to‐fan beam conversion. In: Bruhn A, ed. Pattern Recognition, 40th German Conference; 2018.

[22] Adler J , Kohr H , Oktem O . Operator discretization library (odl), Software 2017. Available from https://github.com/odlgroup/odl.

[23] Abadi M , Barham P , Chen J , et al. Tensorflow: a system for large‐scale machine learning. In: OSDI. Vol. 16; 2016:265–283.

[24] van Aarle W , Palenstijn WJ , Cant J , et al. Fast and flexible x‐ray tomography using the ASTRA toolbox. Opt Express. 2016;24:25129–25147. 27828452 10.1364/OE.24.025129

[25] Gürsoy D , De Carlo F , Xiao X , Jacobsen C. Tomopy: a framework for the analysis of synchrotron tomographic data. J Synchrotron Radiat. 2014;21:1188–1193. 25178011 10.1107/S1600577514013939 PMC4181643

[26] Maier A , Hofmann HG , Berger M , et al. CONRAD–a software framework for cone–beam imaging in radiology. Med Phys. 2013;40:111914. 24320447 10.1118/1.4824926 PMC3820625

[27] Syben C , Seitz S , Maier A. Pyconrad. Software; 2017 Available from https://git5.cs.fau.de/PyConrad/pyCONRAD

[28] Galigekere RR , Wiesent K , Holdsworth DW. Cone‐beam reprojection using projection‐matrices. IEEE Trans Med Imaging. 2003;22:1202–1214. 14552575 10.1109/TMI.2003.817787

[29] Scherl H , Keck B , Kowarschik M , Hornegger J. Fast GPU‐based CT reconstruction using the common unified device architecture (CUDA). In: *Nuclear Science Symposium Conference Record, 2007* NSS’07. IEEE, Vol. 6. IEEE; 2007:4464–4466.

[30] Shepp LA , Logan BF. The fourier reconstruction of a head section. IEEE Trans Nucl Sci. 1974;21:21–43.

[31] Kak AC , Slaney M . Principles of Computerized Tomographic Imaging. New York:IEEE Press; 1988.

[32] Feldkamp LA , Davis L , Kress JW. Practical cone‐beam algorithm. JOSA A. 1984;1:612–619.

[33] Syben C. Code for technical note: Pyro‐nn: Python reconstruction operators in neural networks. Code Ocean; 2019 10.24433/CO.1164752.v1 PMC6899669 31389023

[34] Zeng G , Gullberg GT. Unmatched projector/backprojector pairs in an iterative reconstruction algorithm. IEEE Trans Med Imaging. 2000;19:548–555 11021698 10.1109/42.870265 PMC5297459

