# Supplementary material for: Technical Note: PYRO‐NN: Python reconstruction operators in neural networks
Source: Med Phys. 2019 Aug 27;46(11):5110–5. doi: 10.1002/mp.13753 (PMC6899669; doi:10.1002/mp.13753)
Supplement: Supplementary file 1 — Appendix S1: Supplementary Material. [file MP-46-5110-s001.pdf]

Supplementary Material to
PYRO-NN: Python Reconstruction Operators in Neural Networks

Christopher Syben, Markus Michen, Bernhard Stimpel, Stephan Seitz, Stefan Ploner, and Andreas K. Maier
Pattern Recognition Lab, Friedich-Alexander Universität Erlangen-Nürnberg, 91058 Erlangen, Germany.
(Dated: August 14, 2019)

I. SOFTWARE DESIGN / RATIONALE

The *PYRO-NN-Layers* implementations provide the pure CUDA kernel and the respective control class according to the Tensorflow API. To ensure a robust version control, the framework is directly included into the building process of the Tensorflow sources. In Fig. 1 the process is shown. We provide a patch for all Tensorflow versions since 1.9 which adjust the build process of the Tensorflow sources such that all C++ and CUDA files in the framework directory are built and included into the Tensorflow Python package under the PYRO-NN namespace. This yields the benefit that the user can define specific version combinations for their purpose. Note that the separated build process suggested by Tensorflow can also be used. The separation of the CUDA kernel and information control allows the support of the proposed framework to be extended to other deep learning frameworks. The support for PyTorch is planned for the future.

The architecture is mainly defined by the API to Tensorflow. In Fig. 2 the abstract concept is visualized. Known operators can be implemented as kernels consisting of a C++ class following the design of the Tensorflow API and the actual implementation of the operator as a kernel. The C++ class ensures the behavior of the kernel as a Tensorflow layer in the neural network. In contrast to other frameworks which wrap the implementation on the Python level, this gives full control over the device resources like memory usage.

The *PYRO-NN-Layers* can be used as normal Tensorflow layers on the Python level. Because of the API defined by Tensorflow the user is able to use the known operators like normal Tensoflow layers with the defined input, output and attributes. A crucial aspect is the registration of the gradient to the respective layer. This allows to invoke the relevant algorithm to compute the gradient with respect to the layer in an efficient way. The provision of the gradient is a necessity to enable a gradient flow through the entire network and, thus, allow fully end-to-end trainable networks with known operators. The high level Python API allows to design helper structures for the respective operator. For the CT context, we provide wrapped layers with automated gradient registration and object oriented classes to feed the known operators the necessary attributes. The class design follows the high level abstraction of Tensorflow and wraps the implementation detail to provide the user an easy and convenient usage while preserving the possibility for the user to modify the wrapped methods.

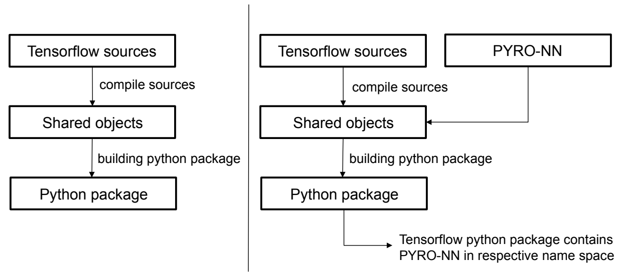

FIG. 1. The build process. Left the build process defined by Tensorflow, right our adjusted process.

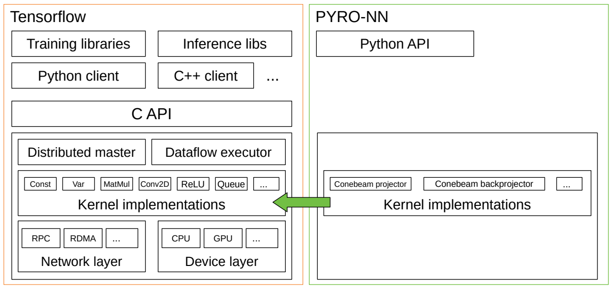

FIG. 2. The architecture of PYRO-NN beside to Tensorflow. Image adjusted from https://www.tensorflow.org/guide/extend/architecture .

All together, these rationals offer the community with a generic, version stable, framework to easily include known operators into neural networks. The source code is publicly available under the Apache 2.0 licence to be directly compatible with Tensorflow and to allow an uncomplicated integration of the community into existing projects.

II. EXAMPLE APPLICATIONS

We show four baseline experiments to outline the capability of the provided framework. This provides the code base for an easy entry into deep learning-based signal reconstruction and to support comparability within emerging methods.

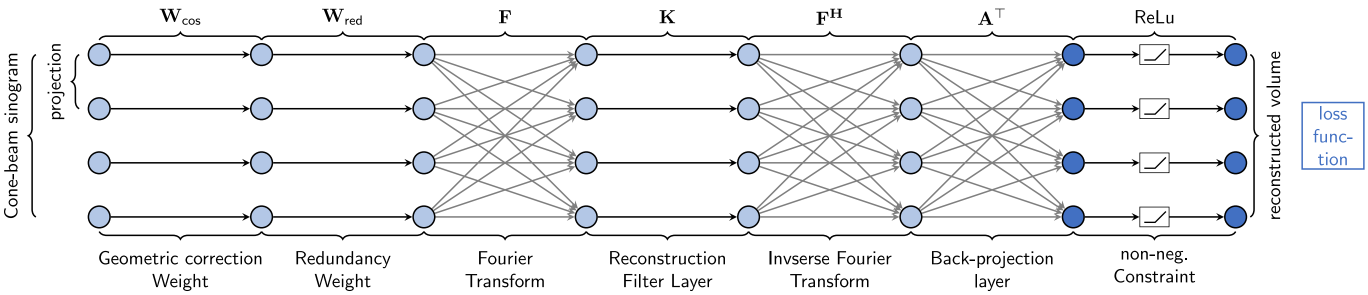

FIG. 3. FDK-Reconstruction Network. Light blue nodes represent the projection domain, while dark blue nodes stands for volume domain.

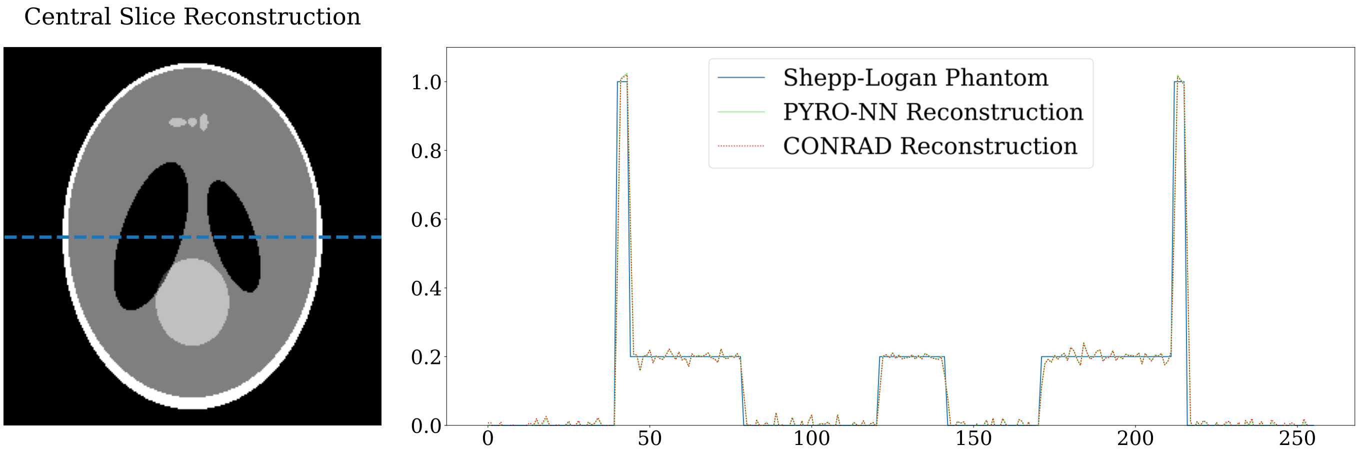

FIG. 4. Short scan FDK reconstruction. The central slice reconstruction shows the result of the network, comparing line plots from phantom (blue), PYRO-NN reconstruction (green) and reference CONRAD reconstruction (red). The gray-level window is [0;0.4].

A. FDK Reconstruction Network

As a proof of concept and validation of the method we provide a FDK reconstruction with Parker weights [1] for a short scan geometry formulated as neural network using the proposed Framework. On the one hand, this setup serves to demonstrate the ability of the presented framework to perform a cone-beam reconstruction. On the other hand, the basic network is a good starting point which makes it possible to integrate further deep learning tools in projection or reconstruction domain. The whole pipeline can then be trained in an end-to-end fashion. This allows general data-driven optimization as well as task specific solutions. Note that the error e.g. ℓ2-loss is computed in the reconstruction domain and can be used to update weights applied in the projection domain. The architecture is shown in Fig. 3. The operation of the FBP algorithm for cone-beam short-scan geometry can be described with

$$\mathbf{x} = \mathbf{A}^{\top}\ \mathbf{F}^{\mathbf{H}}\ \mathbf{K}\ \mathbf{F}\ \mathbf{W}_{red}\ \mathbf{W}_{cos}\ \mathbf{p}\ , \qquad (1)$$

where $\mathbf{W}_{cos}$ is a diagonal matrix describing the pixel-wise independent weighting of the projection data $\mathbf{p}$ with cosine weights. For so called short-scan trajectories some rays are measured twice and some only once. These redundancies will lead to streaking artifacts in the reconstruction if the projection data is not adequately weighted. $\mathbf{W}_{red}$ weights the projection data appropriately, e.g., for a defined cone-beam short scan the weights by Parker [1] can be used. $\mathbf{A}^{\top}, \mathbf{K}, \mathbf{F}$ and $\mathbf{F}^{\mathbf{H}}$ are the system matrix, reconstruction filter and Fourier transform as described in the main paper. The described network architecture can reconstruct a 200 degree cone-beam CT short-scan with 248 projections properly. In Fig. 4 the reconstruction result of a 3D Shepp-Logan phantom is shown. The line profiles illustrate that the result follows the phantom and the reference FDK reconstruction using CONRAD [2].

This architecture can be seen as a baseline, which can be used to insert deep-learning features and non-linearities in the projection as well as in the reconstruction domain while the gradient can be propagated through the whole network. For example, the redundancy weights can be learned for arbitrary short-scan

angles. The update step for the weights according to the backpropagation algorithm is shown by Würfl et al. [3].

B. Real Data Reconstruction

The provided geometry classes as well as the layers are able to reconstruct data from real systems. For fan-beam reconstruction the primary angles stored in the DICOM header can be used to restore the trajectory. For the cone-beam case the projection matrices can be used directly. In Fig. 5 the central slice reconstruction of a pumpkin is shown. The log-transformed raw data of the detector serve as input for the network (Axiom-Artis Siemens Heathineers, Forchheim, Germany). The pumpkin was acquired with a short scan trajectory.

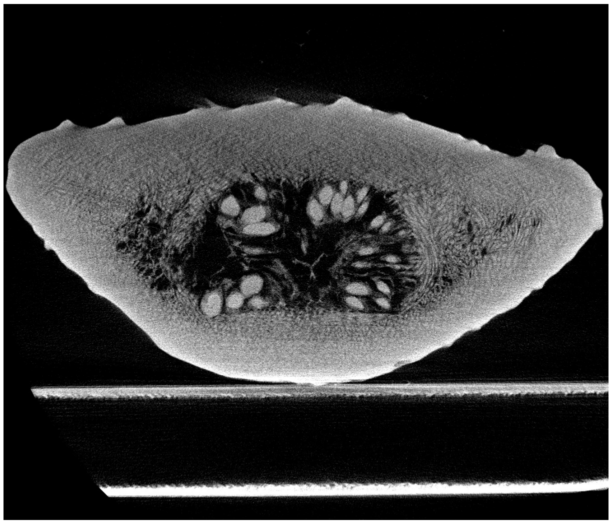

FIG. 5. Short scan reconstruction of a pumpkin from raw detector data. The central slice reconstruction shows the result of the network. The gray-level window is [0;0.7].

C. Learn Reconstruction Filter

To highlight the capability to propagate the gradient through the whole network, from reconstruction domain to projection domain, we demonstrate that the framework can be used to learn the discretized version of the analytical derived ramp-filter for reconstruction in an end-to-end network approach.

The network architecture is defined by

$$\mathbf{x} = \mathbf{A}^{\top}\ \mathbf{F}^{\mathbf{H}}\ \mathbf{K}\ \mathbf{F}\ \mathbf{p}\ , \qquad (2)$$

where $\mathbf{K}$ is the reconstruction filter initialized with the ramp filter and the only trainable layer in the network. For an filtered back-projection algorithm the ramp filter applied in the projection domain is the continuous analytical derived solution. Without proper discretization of the filter, offset and cupping artifacts will occur in the reconstructed volume. The proper discretization is the so called RamLak filter [4].

We can show that we can use the backpropagation of the network to compute the error on the reconstruction domain and adjusting the filter weights of $\mathbf{K}$ in the projection domain. The result is converging towards the analytical derived discretized RamLak filter as shown in Fig. 6. A detailed description and discussion of the experiment is given by Syben et al. [5]. Note that the training of the filter was done with purely numerical data and can directly applied on real data without loss of validity.

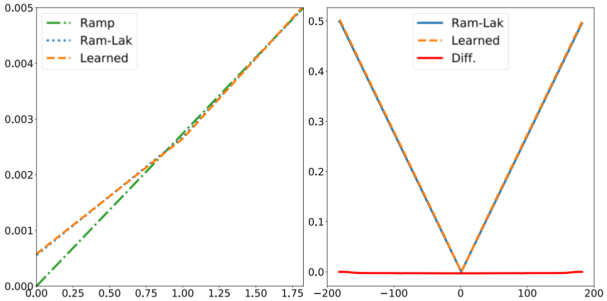

FIG. 6. Ramp, RamLak and Learned filter.

D. Iterative Reconstruction

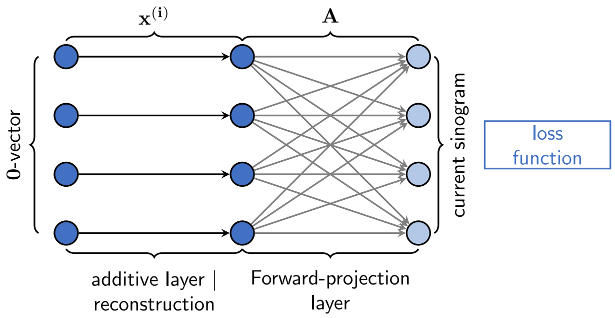

FIG. 7. Iterative reconstruction network. Light blue nodes represent the projection domain while dark blue nodes stand for volume domain.

With the third experiment we want to demonstrate the versatility and possibilities which are offered by the framework. We can model a network such that an iterative reconstruction algorithm with regularizers can be conducted. The network is designed such that the backpropagation algorithm mimics the iterative reconstruction based on a data fidelity term. Starting with the data fidelity term for iterative reconstitution with total variation (TV) regularizer

$$\min\ ||\mathbf{A}\mathbf{x} - \mathbf{p}||_2^2 + \lambda \mathbf{TV}(\mathbf{x}) \qquad (3)$$

where $\mathbf{A}$ is the system matrix, $\mathbf{x}$ is the volume to be reconstructed initialized with zero and $\mathbf{p}$ is the measured

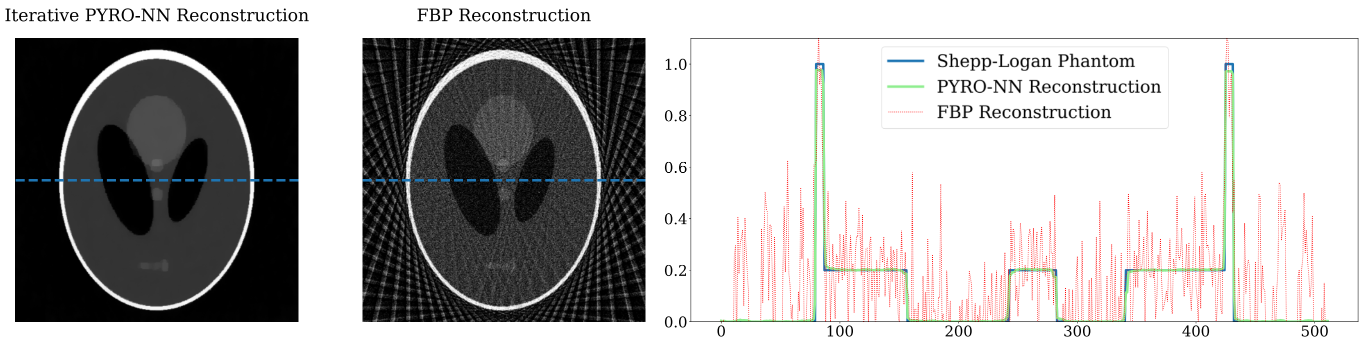

FIG. 8. Sparse view reconstruction. The reconstruction shows the result of the iterative reconstruction network, comparing line plots from phantom (blue), iterative PYRO-NN reconstruction (green) and reference FBP reconstruction (red). The gray-level window is [0;1.0].

sinogram. The data fidelity term can be also interpreted as a network architecture, where the input of the network is a zero-vector, followed by an additive layer and then forward projected using the provided forward projection layer as shown in Fig. 7. The loss function is then the $\ell 2$-loss w.r.t to the measured data $\mathbf{p}$. Training the network will update the weights of the additive layer. Thus, the seeked reconstruction is represented by the weights of the additive layer at the end of the learning process.

We demonstrate that the network setup can reconstruct a sparse view CT acquisition with 30 parallel projections over an angular range of 180 degree. 2% additive Gaussian noise where applied to the projection data. In Fig. 8 the reconstruction with the iterative PYRO-NN reconstruction is shown and compared to an FBP reconstruction and the Shepp-Logan phantom. Note that the actual TV-regularized iterative reconstruction using the proposed framework needs only few lines of code. The computation of TV is provided by Tensorflow. With this baseline example we provide an easy-to-use starting point for future investigations into learned regularizers and different loss functions.

ACKNOWLEDGMENTS

The research leading to these results has received funding from the European Research Council (ERC) under the European Unions Horizon 2020 research and innovation program (ERC Grant No. 810316). Additional financial support for this project was granted by the Emerging Fields Initiative (EFI) of the Friedrich-Alexander University Erlangen-Nürnberg (FAU).

[1] D. L. Parker, Optimal short scan convolution reconstruction for fan beam ct, Medical physics **9**, 254 (1982).
[2] A. Maier, H. G. Hofmann, M. Berger, P. Fischer, C. Schwemmer, H. Wu, K. Müller, J. Hornegger, J.-H. Choi, and C. Riess, Conrad – a software framework for cone-beam imaging in radiology, Medical physics **40** (2013).
[3] T. Würfl, M. Hoffmann, V. Christlein, K. Breininger, Y. Huang, M. Unberath, and A. K. Maier, Deep learning computed tomography: Learning projection-domain weights from image domain in limited angle problems, IEEE transactions on medical imaging **37**, 1454 (2018).
[4] G. Ramachandran and A. Lakshminarayanan, Three-dimensional reconstruction from radiographs and electron micrographs: application of convolutions instead of fourier transforms, Proceedings of the National Academy of Sciences **68**, 2236 (1971).
[5] C. Syben, B. Stimpel, K. Breininger, T. Würfl, R. Fahrig, A. Dörfler, and A. Maier, Precision learning: Reconstruction filter kernel discretization, in *Proceedings of the Fifth International Conference on Image Formation in X-Ray Computed Tomography* (2018) pp. 386–390.
